# Characters of Broussais and Larrey

**Published:** 1846-10

**Authors:** 


### SELECTIONS

FROM

### AMERICAN AND FOREIGN JOURNALS.

*Characters of Broussais and Baron Larrey.* By Reveillé-Parise. *Broussais.*—Entering life amidst the turbulence of the French Revolution, the early part of the career of Broussais was stormy and uncertain. Soldier, corsair, hospital-clerk, navy-surgeon, student, civil practitioner, and lastly, military surgeon in the Napoleon campaigns, his existence was destitute of repose. Nevertheless, robust in body, firm in determination, and gifted with great aptitude for work, he pursued his studies in spite of every fatigue and difficulty. He eventually came to Paris and became a pupil and an in timate of Bichat, and an ardent admirer of the opinions of Pinel. His treatise *Des Phlegmasies Chroniques*, which had cost him immense labor, fell almost still-born from the press; but, although he felt the disappointment in the keenest manner, he concealed it, and pursued his indefatigable researches in Spain and Italy, examining thousands of bodies, accumulating a vast mass of facts, and meditating over them for years.

It was in 1815 that he commenced his private course. It was only two paces from the Faculty that he cast forth his haughty defiance and raised altar against altar, doctrine against doctrine. From the first he announced himself as a reformer of science, proceeding by physiological demonstration to a complete rebuilding up of the structure of medicine. And let it not be supposed his course resembled any other of this kind, that is a simple and calm exposition, methodically elaborated, of the principles of the art. No, it was literally a true arena where the professor fought alone and to the death. It was on his part an exhibition of harshness and frankness, an aggressive and ill-boding discussion of received theories, a continual explosion of recrimination against the science of

6*

formerly and its constructors, a constant affirmation of his own doctrines, a haughty and disdainful treatment of those of others—the like of which had never yet been seen. Then, the choleric argument, the passionate tone, abrupt gestures, the thundering voice of the professor, and his animated countenance, for he always seemed as if illumined with inspiration, added much to the effect of his addresses. The numerous and crowded auditory kept the deepest silence, the more as their attention was always, so as to say, enslaved, because it was continually excited. * * * * Broussais joined to a great penetration a very cultivated mind; and thus we may remark in his lectures, in his reiterated appeals to the progress of science, a skilful mixture of truth and error, a certain art of presenting the latter with the probabilities of reality. Although exclusive in his ideas he announced solid principles. Not content with observing and collecting many facts, he knew how to compare them, and deduce from them consequences often strained, but at other times undoubtedly just. To consider science under aspects until then unknown; to open a new horizon to ulterior researches in the study of diseased organs; to state his principles in an exact and precise manner; to reduce them to the rigor of a theorem; such was the end he proclaimed, and which he flattered himself he attained. So much knowledge and application, such high pretensions, united to the fire of conviction which seemed to animate him, to that ardor of proselytism, to that perseverance in self-glorification, to that fanatical esteem of his system, perpetually announced as the complete *resumê* of the entire range of medical truths, gave to the lectures of Broussais an astonishing popularity. Auditors crowded and pushed their way to them as to a theatrical amusement, and, more than once, pupils, who were unable to gain admission, might be seen taking their notes in the sun or rain when the loud tones of the professor allowed them to seize the purport of some of his observations.

In 1815 his *Examen* appeared. In this, the opinions of his adversaries were handled with the bitterest sarcasm and irony, and his own doctrine developed with surpassing skill. His success was prodigious. Not only were his dogmas received with enthusiasm by the schools, but even the convictions of elder practitioners were much shaken; so that the advent of a new era of medicine was proclaimed on every side. The deceptive simplicity of the system, and the magical words "progress," "advancement of science," "new doctrine," contributed immensely to its popularity; and those who dared to attempt to oppose its reception were covered with terms of reproach and contempt. It is not surprising that Broussais became intoxicated with the incense constantly poured out before him, and imagined himself exalted into supreme judge of what should or not be received as sound doctrine. Yet, at the end of a few years, it was discovered that he, like many other reformers, was more potent in criticizing and destroying present structures than in raising new ones; and that the system of the man who had been so ready of visiting those of others with sarcasm and contempt, was not itself proof against the shafts of the critic. Remarks, criticisms, exceptions, and objections now multiplied on every side; but we need not follow the exposure of the errors of a system which never obtained much currency here, notwithstanding the Gallomania which some of our Journals endeavored to foster. M. Parise exhibits concisely not only the unscientific character of the principles laid down, but the bad effects which too frequently flowed from their practical adoption; for a practice which had been borne by the robust young soldiers coming within the sphere of Broussais' observation, was ill-suited to the feebler constitutions so often met with in civil practice. Once submitted to a rational examination, the system rapidly declined in estimation; and even when in the possession of an official professorship its constructor could not raise it from the neglect and disfavor into which it had fallen. His vehement denunciations against the older systems no longer drew the crowded auditories of heretofore, and the few pupils who attended his lectures seemed to do so more as a curiosity of by-gone days than as a reality of the present. Whether from faith in its truth, or from the desire of drawing upon himself the attention which had so notoriously flagged, Broussais next became a propagator of phrenology. Again he had a large and applauding, if not a judicious auditory: "but the concessions he made to his adversaries were so numerous, that Gall would hardly have recognized his own doctrines in his discourses." He adopted for the defence of this the same paradoxical arguments, the mixture of error and truth by which he had sought to defend the doctrine of irritation. It will not seem surprising that Broussais only attained a rank in the practice of the profession very disproportionate to his merits and reputation. His exclusive mode of viewing cases so limited his range of therapeutical indications, although the force of circumstances sometimes obliged him to make concessions. One of those related of him is amusing enough. A patient having declared to him that he could support his regimen no longer, and that he was literally dying of hunger, Broussais reflected for a moment and replied, "Very well, you carnivorous animal, I will satisfy you," and ordered him a spoonful of broth in a glass of water. However, concessions of any kind were wrung from him with difficulty, for he prided himself upon his inflexibility to objections or adversaries. Nevertheless, after venting the most bitter sarcasms on places and riches, he allowed himself to accept some of these, and the great agitator was found lecturing in the professor's gown, and placing six lines of honourable titles after his name in one of his works!

Regarding Broussais with impartiality, uninfluenced by the excessive praise of his admirers or the bitterness of his detractors, we shall find he was a man of rare capacity and incontestible merit. It will be allowed that he has rendered services to science and has proclaimed truths which will endure. Nevertheless, if he appears great, the narrowness of his views is striking when compared with those of Bichat. This is because he was too exclusive, too absolute, in his ideas, because he has forcibly referred facts to two or three principles only, that is to either too few or too many, and because he has, with a view to his system, proclaimed the true and the false with an equal ardor. In the place of pruning, adding, and extending, he was desirous of radically destroying and re-constructing; and, at a period when doubts were raised, he struggled for the most formal materialism. Thus, although he has shed a luminous ray on science, although he proclaimed himself its master, his opinions have been attacked, his pedestal shaken; and his titles to true glory strongly contested. What remains of him now? Has Broussais done what he ought and could? Has he fulfilled his mission worthily and completely? Posterity, that high court of appeal for reputations, will decide in time to come. She will be just, yet severe; for, according to Scripture, 'much is required of him to whom much is given.'

*Larrey.*—The character and services of Larrey are so well known and appreciated in this country, that we need borrow little from the sketch here drawn by his friend and colleague. It is always, however, pleasing to accumulate instances of the excellent conduct of the members of our profession; and we question if the *beau-ideal* of a military surgeon was ever more realized than in the character of this celebrated man.

At the battle of Eylau he remained 30 hours without eating; and so hurried and ardent was he in dressing wounds and giving orders, that paralysis of the bladder was nearly induced. Friends, enemies, every one, might claim his services. He saw only suffering creatures imploring the succor of art. Degree of rank in nowise decided his preferences. No matter what the epaulette, he only saw the wounded man, whose blood, poured out for his country, was equally noble and precious. After having operated on Marshal Lannes, and visited Duroc, the intimate friend of Napoleon, he would dress with equal eagerness the wounds of the lowest soldier or the conscript of yesterday. His colleagues even, always his dearest friends, never met with preference. Our hononable *confrère*, M. Tanchou, tells us that, when wounded in the battle of Montmirail, he was taken to Larrey's *ambulance:* 'Your wound is a slight one,' he said, 'we have only room and straw here for bad accidents. However, they may put you in yonder stable.' The gravity of a wound was in fact the only ground of preference in his eyes. * * * * * * * The more severe and doubtful an action became, the more the activity and resources of this great surgeon multiplied—his first and only thought being the succor of the wounded. During the highest raging of the battle of Waterloo, when the fire was continuous and murderous, the author of this sketch and Larrey met, when he exclaimed, 'My dear colleague, look to your wounded soldiers. Pay attention only to them.' He added, 'the firing is hot indeed, but let every one do his duty and all will go on well. * * * * * Larrey had so little dissimulation, that one of his defects, perhaps the only one, was the high opinion he entertained of his own talent and works. He praised himself with such a good faith and confidence, but his weakness was the more excusable, as it was not without foundation, facts answering to words. Frequently a deep colossal pride, peculiar to celebrated men, is disguised without, and hidden with care; but Larrey concealed nothing. All his thoughts and words were without disguise, for he knew not how to conceal his pride at the bottom of his heart, like some who are termed modest. Oftentimes he raised a pedestal to himself with the same simplicity, the same confidence, as if he had performed the most virtuous action or the greatest sacrifice. We may sometimes smile at his simple and frank want of modesty, and at the same time admire his noble heart full of rectitude, honor, probity, and courage, a stranger to the manœuvres of personal interest, concealed under the veils of philanthropy and love of science. What he delighted in, above all things, was to protect and assist others by his counsels and his credit; so that he always regarded surgeons whom he had known, and who were a little younger than himself, as his pupils. In writing to the late M. Coutanceau, then professor at *Val-de-Grâce*, Member of the Academy, and author of different works, he never omitted concluding with 'your affectionate brother-in-law and former professor.' This was not pride or the desire of superiority, but a bond of patronage, good-will, and reciprocal attachment, which he delighted in tightening and continuing.'

In perusing the accounts of the lives of late and present members of our profession in France and in our own country, we have often been struck with the far larger proportion in the former nation who have raised themselves from indigence and comparatively low conditions to the highest position of professional eminence. And in the facilities which that country affords to her industrious and enterprising youth, destitute of family connections, pecuniary resources, or hospital influences, for attaining the highest offices in her power to bestow, we see an example well worthy of imitation in our own country, where a very different system of selection, or rather of succession, prevails.—*Med. Chir. Rev.*

